# Changes in modifiable risk factors in women at increased risk for breast and ovarian cancer during the COVID-19 pandemic

**DOI:** 10.1016/j.heliyon.2024.e35417

**Published:** 2024-07-30

**Authors:** Kathrin Stewen, Annika Droste, Christian Ruckes, Tania Elger, Susanne Theis, Anne-Sophie Heimes, Mona Wanda Schmidt, Lina Judit Schiestl, Philip Herbert Klecker, Katrin Almstedt, Marcus Schmidt, Walburgis Brenner, Annette Hasenburg, Roxana Schwab

**Affiliations:** a Department of Obstetrics and Gynecology, University Medical Center of the Johannes Gutenberg University Mainz, Langenbeckstrasse 1, 55131, Mainz, Germany; b Interdisciplinary Center for Clinical Trials Mainz, University Medical Center of the Johannes Gutenberg University Mainz, Langenbeckstrasse 1, 55131, Mainz, Germany; c Department of Obstetrics and Gynecology, University of Tuebingen, Calwerstraße 7, 72076, Tübingen, Germany

**Keywords:** Modifiable lifestyle factors, Breast cancer, Ovarian cancer, COVID-19 pandemic, Physical activity

## Abstract

**Background:**

Modifiable lifestyle factors exert a substantial influence on the development of various diseases. The COVID-19 pandemic necessitated the implementation of containment measures to mitigate the viral spread, which affected the maintenance of healthy habits.

**Methods:**

Changes in lifestyle factors (e.g. physical activity, nutrition, smoking, drinking alcohol) within a cohort of German women at increased risk of breast cancer (BC) or ovarian cancer (OC) were evaluated through an anonymous web-based survey. The self-reported assessment of mental health was conducted using the PHQ-4 questionnaire. This tool combines two items from the Patient Health Questionnaire for Depression (PHQ-2) and two queries from the Generalized Anxiety Disorder Scale (GAD-2). Potential predictors of lifestyle changes were determined via multiple logistic regression analysis. A heuristic model was employed to project potential long-term consequences on BC incidence.

**Results:**

During the pandemic, 41.6 % of respondents reported reduced engagement in physical activity (PA), whereas 14.3 % reported increased engagement in PA. A score ≥5 on the PHQ-2 scale emerged as an independent risk factor for reduced PA (OR 12.719; 95 % CI 1.089–148.549; p = 0.043). By the heuristic approach, we projected an increase of BC by 3384 cases in Germany by 2030, which is attributable to the alterations in PA patterns during the pandemic.

**Discussion:**

Impaired mental health during the pandemic constituted a risk factor for unfavorable changes in PA. Consequently, a surge in BC may arise due to decreased engagement in PA. Healthcare professionals must remain aware of the potential risk factors that facilitate adverse alterations in modifiable risk factors caused by pandemic-related contingency measures or similar future events.

## 1 Introduction

In late 2019, initial reports emerged concerning an infectious disease outbreak in China. The newly identified SARS-CoV-2 virus exhibited rapid global dissemination within weeks [1,2], resulting in a significant proportion of cases with a severe clinical course and increased mortality rates, particularly among vulnerable populations [[3], [4], [5]].

To mitigate the virus's spread and avert severe outcomes linked to SARS-CoV-2 infection, particularly within high-risk groups like the older adults and individuals with preexisting medical conditions, governments worldwide enacted an array of social distancing measures. These measures entailed curtailing or prohibiting social activities, such as group exercise, and shuttering facilities like fitness centers and community sports venues. The stringent limitations, coupled with the psychological distress caused by the pandemic, fostered sedentary and maladaptive behaviors and engendered other unhealthy lifestyle factors, including unfavorable dietary habits and elevated consumption of alcohol and nicotine on a global scale [[6], [7], [8], [9], [10]]. A decline in physical activity (PA) was reported across various nations in Europe and worldwide, including the general population and those with preexisting medical conditions [11,12].

In Germany, as of March 22, 2020, public gatherings exceeding two individuals who did not share a household were prohibited. The German government encouraged people to engage in solitary physical exercise since sporting facilities were temporarily closed or operated with restricted access, and public sports events were canceled [13,14]. A quantitative assessment of average step counts over a one-week period for users of Fitbit© devices comparing March 2020 to March 2019 revealed a decrease of up to 38 % and 25 % in average steps in Spain and Italy, respectively, during the week ending March 22, 2020 [15]. Furthermore, Germany had a 7 % reduction in average steps during the same time frame [15]. A cross-sectional, representative, web-based survey conducted among the German population reported a 31.1 % decrease in PA until the end of April 2020 compared to pre-pandemic leisure-time PA [7].

Modifiable lifestyle factors, including PA and dietary choices, play pivotal roles in the development of various non-communicable diseases, such as cardiovascular disease and diabetes, as well as certain malignancies [16,17]. Lifestyle factors also exert a notable influence on cancer-related events, including recurrence and mortality, across a range of cancer types, such as breast, colorectal, pancreatic, and lung cancers [[18], [19], [20], [21], [22]].

Physical exercise has been acknowledged as a primary preventive factor in breast cancer (BC), as it is associated with a reduced incidence, lower recurrence rates, and decreased mortality in BC patients [18,[23], [24], [25], [26], [27], [28]]. In the German population, as of 2018, an estimated 27,081 cancer cases could be attributed to low levels of PA [18]. It is important to note that a direct, specific cause-effect mechanism does not characterize the relationship between lifestyle factors and cancer, but rather involves a complex interplay of multiple pathways in carcinogenesis [20].

A sedentary lifestyle stands out as a leading global contributor to mortality, accounting for 6 % of worldwide deaths, and prolonged periods of physical inactivity are associated with an increased overall risk of cancer by up to 20 % [22]. Excessive caloric intake, imbalanced dietary patterns, and weight gain trigger metabolic alterations, including insulin resistance, up-regulation of insulin-like growth factors, modification in sex hormone metabolism, and chronic inflammation, collectively elevating the susceptibility to several cancer types [20].

Dietary choices seem to facilitate cancer development by promoting resistance to apoptosis, supporting angiogenesis, and contributing to mechanisms of tissue evasion, metastasis, and evading immune surveillance [20]. This has been linked to a 20 % increase in the risk of BC. In comparison, a significant increase in red meat consumption is associated with a 3 % increase in BC incidence. A decrease in dietary fiber intake is correlated with a 5 % increased incidence of BC [18].

### 1.1 Aim of the study

For the first time in decades, entire societies implemented widespread restrictions on healthy activities to curb the spread of the novel SARS-CoV-2 virus, which posed an imminent and incompletely quantifiable threat to public well-being. We hypothesized, that the COVID-19 pandemic negatively influenced the lifestyle of people. This study aimed to assess the lifestyle modifications and potential influencing factors among German women at an elevated risk for BC and ovarian cancer (OC) during the initial year of the COVID-19 pandemic. Additionally, the research sought to quantify the impact of lifestyle changes, particularly in PA, on BC incidence using a heuristic approach.

## 2 Materials and methods

### 2.1 Study population

The web-based survey was active from January 29 to April 22, 2021. The survey was made accessible through a direct link and distributed as an “open survey” via 10.13039/100005801Facebook platforms of patient support groups for women at increased risk of breast cancer (BC) and ovarian cancer (OC). The invitation to participate in the study was posted once. Those who were members of the respective platform and accessed the platform during recruitment had a chance to participate in the survey, and no individual or personalized invitations were sent. Data collection was performed anonymously using the SoSciSurvey platform, which adheres to stringent EU data protection laws, with no collection of personal information such as IP addresses.

Participation was voluntary, and no incentives were provided to study participants. Inclusion criteria comprised individuals aged 18 years or older, self-reported elevated risk for BC or OC (including women with a prior malignant diagnosis of BC or OC and those at increased risk without a history of malignant disease), and informed consent to participate. These inclusion criteria were assessed in the initial section of the survey, and only those who met these criteria were eligible for participation.

The information about increased risk for BC and OC was self-reported, and no medical confirmation or medical records were required. The following questions assessed the increased risk for BC and OC:

“To which risk group do you belong:

- I was diagnosed with a mutation in the BRCA1 or BRCA2 gene
- I was diagnosed with a different mutation (except BRCA1 or BRCA2 gene)
- I have an increased risk due to my family history, but I wasn't diagnosed with a gene mutation (yet)”

The survey questionnaire included queries related to demographic characteristics (age, partnership status, living arrangements, and educational level), disease-specific factors (e.g., history of BC or OC), and pandemic-related variables (e.g., COVID-19 history, reduction in social contacts) parameters, mental health and resilience. The specific questionnaire designed for this study is available as supplement material. The choice of measures used to assess the changes in modifiable lifestyle factors was based on thorough literature research on the respective topic. Changes in modifiable lifestyle factors were assessed using a 6-point Likert scale (significantly less - less -unchanged - more/increased – significantly more/increased - does not apply) in response to the following question:

“How has your lifestyle changed in the last 12 months (during the global COVID-19-pandemic) in terms of:

1. Consumption of alcohol;
2. Smoking;
3. Exercising regularly;
4. Effort to achieve/maintain a healthy body weight;
5. Following a balanced diet and avoiding a high-caloric diet;
6. Avoidance of/reduction in red meat consumption.”

The mental health of the study participants was evaluated using the Patient Health Questionnaire for Depression and Anxiety (PHQ-4). The PHQ-4 is a comprehensive screening tool for depression and anxiety, comprising two items from the Patient Health Questionnaire for Depression (PHQ-2) and two items from the Generalized Anxiety Disorder Scale (GAD-2) [29].

PHQ-2 and GAD-2 scores equal or greater than ≥3 were considered as cut-off points (“yellow flag”), indicating a high likelihood of depressive or anxiety disorders, as participants scored higher than 93 % of the normative general population. Major depressive disorders were predicted with a sensitivity of 82.9 % and a specificity of 90.0 % when PHQ-2 ≥3 was employed [30]. Generalized anxiety disorder was predicted with a sensitivity of 86.0 % and a specificity of 83.0 % when GAD-2 ≥3 was utilized [31]. Scores of PHQ-2 and GAD-2 scores of ≥5 were designated as “red flags” (indicating a very high probability for depressive or anxiety disorders, as participants scored higher than 99 % of the normative general population) [29,32]. Resilience was assessed using the Brief Resilience Scale (BRS) [33].

### 2.2 Heuristic approach to assess the impact of lifestyle changes on breast cancer incidence

In this study, we adopted a heuristic approach to evaluate the potential influence of lifestyle changes on the incidence of BC. The heuristic approach was chosen, as directly measuring the long-term impact of lifestyle changes on cancer incidence is complex and requires longitudinal data over many years or even decades. Given the recent nature of the COVID-19 pandemic, there isn't sufficient direct empirical data available to assess its impact on cancer incidence through lifestyle changes immediately. A heuristic approach allows for estimating potential impacts based on existing data and well-established relationships between lifestyle factors and cancer risk. By using relative risk (RR) values and data on lifestyle changes during the pandemic, this study can estimate the potential increase in BC incidence. This method involves applying known RRs to the observed changes in behavior among the study population to predict future incidence rates, a process well-suited to heuristic analysis.

Cancer registry data from the Robert Koch Institute, including the German Centre for Cancer Registry, was obtained through a database query [34,35]. Due to the relatively low baseline incidence of OC, our analysis focused solely on the impact on BC incidence.

To analyze the changes in cancer incidence, we employed the projection developed by Quante et al. [36], which estimated 91,200 projected cases (PC_2030_) of BC among women in Germany in the year 2030, taking into account expected demographic changes. We further calculated the projected cases for the year 2030 after factoring in the effect of the COVID-19 pandemic on PA (PC_2030_PA_) using the following formula:

PC_2030_PA_ = PC_2030_ x (P_H_ΔPA_/RR_Incidence_PA_)+(1- P_HΔPA_)).

Here, P_H_ΔPA_ represents the absolute percentage of healthy persons' decreased PA during the COVID-19 pandemic, and RR_Incidence_PA_ signifies the RR for BC incidence when comparing persons with high vs. low recreational PA [18]. The excess cases of incident BC cases were computed by the formula ΔBC_incidence_ = PC_2030_PA_ - PC_2030_.

To assess the potential economic impact, we considered the costs of medical treatment for BC at EUR 21,455 per patient in the first 11 months following diagnosis [37], and assumed that these expenses would remain stable for the next 10 years. This amount of costs was multiplied with the excess cases of BC.

### 2.3 Statistics

Descriptive statistics were used to characterize the study population, including frequencies, means, and standard deviations. Spearman correlation, characterized by the correlation coefficient ρ, was utilized to assess the statistical association between two ordinal variables that represented changes in lifestyle factors (with the respective Likert-scale variables grouped as increased unhealthy behavior, unchanged behavior, or decreased harmful behavior).

To identify risk factors contributing to unhealthy behavior, the Likert scale variables were clustered into unhealthy behavior (comprising either less and significantly less or more and significantly more, as appropriate) as the dependent variable. In contrast, unchanged lifestyle factors and healthy behavior (comprising either less and significantly less or more and significantly more, as appropriate) were treated as controls. Univariate analyses were used to identify variables with a robust discriminatory potential for worsening of modifiable lifestyle factors.

Variables with p-values less than 0.25 in the univariate regression model were subsequently entered into the final model multivariate logistic regression using a backward stepwise selection method to ascertain the independence of the aforementioned variables in predicting modifiable lifestyle factors [38,39]. The odds ratio (OR), p-value, and 95 % confidence interval (95 % CI) were employed to express the statistical findings.

All statistical tests were two-tailed, and a significance level of p < 0.05 was applied. The statistical analyses were conducted using SPSS® software Version 27 (IBM Corp.).

## 3 Results

### 3.1 Demographic characteristics of the study group

Out of the 89 participants who at least completed the first page of the questionnaire, a total of 76 respondents provided a response to the questions concerning lifestyle changes during the COVID-19 pandemic. The demographic characteristics of the study group are displayed in Table 1.

**Table 1:** Demographic characteristics of the study group.

**Age**		
	**M (SD); N**	43.54 (10.38); 74
	**Mdn (IQR)**	43.00 (34.75–52.00)
**Having a stable partnership**		
Yes	% (n/N)	92.1 (70/76)
No	% (n/N)	7.9 (6/76)
**Living alone**		
yes	% (n/N)	7.9 (6/76)
no	% (n/N)	92.1 (70/76)
**Education**		
Up to secondary level education	% (n/N)	54.7 (41/75)
Tertiary level education	% (n/N)	45.3 (34/75)
**Did you have COVID-19 disease?**		
Yes	% (n/N)	3.9 (3/76)
No	% (n/N)	96.1 (73/76)
**Someone in your social network having COVID-19**		
Yes	% (n/N)	27.6 (21/75)
No	% (n/N)	72.0 (54/75)
**Reduction in social network**		
None to moderate reduction	% (n/N)	15.8 (12/76)
Strong to a very strong reduction	% (n/N)	84.2 (64/76)
**Having a history of OC and BC (in situ or invasive)**		
Yes	% (n/N)	65.8 (50/76)
No	% (n/N)	34.2 (26/76)
**Psychological variables**		
PHQ-2	Mean (SD); N	2.33 (1.78); 63
	Median (IQR)	2.00 (1.00–4.00)
PHQ-2 ≥ 3	% (n/N)	36.5 (23/63)
PHQ-2 ≥ 5	% (n/N)	9.5 (9/63)
GAD-2	Mean (SD); N	3.71 (1.16); 63
	Median (IQR)	4.00 (3.00–5.00)
GAD-2 ≥ 3	% (n/N)	88.8 (56/63)
GAD-2 ≥ 5	% (n/N)	25.4 (16/63)
PHQ-4	Mean (SD); N	6.05 (2.24); 63
	Median (IQR)	6.00 (4.00–7.00)
PHQ-4 ≥ 6	% (n/N)	50.8 (32/63)
PHQ-4 ≥ 9	% (n/N)	15.8 (10/63)
BRS cv	Mean (SD); N	3.37 (0.72); 63
	Median (IQR)	3.33 (2.83–4.00)

BRS = Brief Resilience Score; PHQ-4 = Patient Health Questionnaire for Depression and Anxiety; GAD-2 = Generalized Anxiety Disorder Scale; PHQ-2 = Patient Health Questionnaire for Depression; N = Number of women for whom data were available; n = sample size; M = mean; SD = standard deviation, Mdn = median; IQR = Interquartile Range; cv = continuous variable.

### 3.2 Changes in lifestyle during the COVID-19 pandemic

**Fig. 1:**
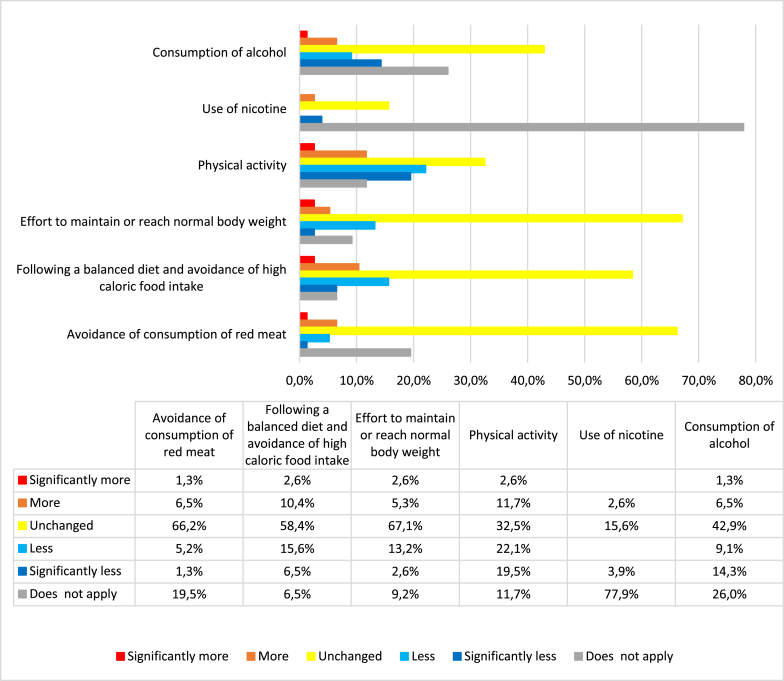
Changes in lifestyle factors during the COVID-19 pandemic (% of respondents).

Fig. 1 illustrates the alterations in lifestyle habits among German women with an increased risk of developing BC and OC during the COVID-19 pandemic. The data reveal that 67.1 % of participants reported no changes in their efforts to maintain or achieve a normal body weight, 58.4 % indicated that they maintained their balanced diet with avoidance of high-caloric intake, and 66.2 % did not alter their consumption of red meat (Fig. 1). Regarding nicotine use, 77.9 % of participants mentioned that it did not apply to them (Fig. 1).

However, the most significant lifestyle changes were observed in physical activity (PA) within the entire study population. Among the participants, 14.3 % reported engaging in more or significantly more PA during the pandemic than before, while 41.6 % noted that they had engaged in less or significantly less PA. Meanwhile, 32.5 % did not modify their PA habits, and 11.7 % did not provide a response (indicated as “does not apply”) to the question (Fig. 1).

To gain deeper insights into changes in PA, specifically among women with BC, the study group was stratified into those with a history of BC (in situ or invasive) and those without a BC diagnosis. Among participants with a BC history, 21.3 % reported an increase in PA, 23.4 % maintained the same level of PA as before the pandemic, and 44.7 % reported a decrease in PA. This resulted in a net excess of 23.4 % of participants with a BC history who engaged in less healthy behavior concerning PA during the pandemic compared to their pre-pandemic habits. A total of 10.6 % (5 out of 50) did not specify their PA status during the pandemic and responded with “does not apply."

**Fig. 2:**
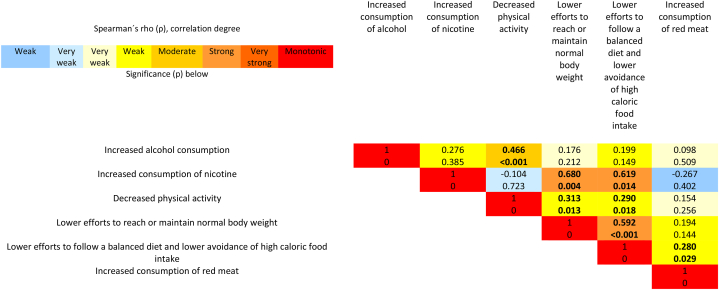
Correlations between health behavior (modifiable lifestyle factors) in the study population. Spearman rank correlation matrix of all variables regarding lifestyle changes during the COVID-19 pandemic in Germany. Values above show the Spearman rank results; values below show the p-values. Significant correlations are in bold, and the significance level was set to p < 0.05. Red colors show positive correlations; blue colors show negative correlations.

When examining the interrelationships between lifestyle changes, it becomes evident that each unfavorable lifestyle alteration exhibited significant correlations with at least one other unfavorable lifestyle change (Fig. 2). Reduced adherence to a balanced diet and a diminished effort to avoid high-caloric foods displayed strong correlations with increased nicotine consumption and a decreased attempt to achieve or maintain a normal body weight. Additionally, these changes exhibited a weak correlation with reduced PA (Fig. 2). Decreased PA demonstrated a moderate correlation with increased alcohol consumption and a weak correlation with a reduced attempt to attain or maintain a normal body weight (Fig. 2).

### 3.3 Possible predictors of lifestyle changes

We conducted univariate logistic regression analyses to explore potential predictors of lifestyle changes (Table 2).

**Table 2:** Possible predictors of modifiable lifestyle factors (univariate logistic regression analyses).

	Increased consumption of alcohol		Increased consumption of nicotine		Decreased physical activity		Lower efforts to reach or maintain normal body weight		Lower efforts to follow a balanced diet and lower avoidance of high caloric food intake		Increased consumption of red meat	
	p	OR (95 % CI)	p	OR (95 % CI)	p	OR (95 % CI)	p	OR (95 % CI)	p	OR (95 % CI)	p	OR (95 % CI)
Age ≥51 (years)	0.201	3.083 (0.548–17.352)	n.a.	n.a.	0.144	2.160 (0.769–6.073)	0.612	1.391 (0.389–4.973)	0.882	1.091 (0.347–3.426)	0.975	1.030 (0.159–6.670)
Having a stable relationship (co: not having a stable relationship)	n.a.	n.a.	n.a.	n.a.	0.381	2.818 (0.278–28.553)	n.a.	n.a.	0.678	1.600 (0.174–14.726)	n.a.	n.a.
Living alone (co: not living alone)	n.a.	n.a.	n.a.	n.a.	0.880	1.138 (0.213–6.083)	0.189	3.600 (0.532–24.365)	0.562	1.700 (0.283–10.206)	0.235	4.500 (0.377–53.769)
Tertiary level education (co: up to secondary education level)	0.501	1.846 (0.310–11.011)	n.a.	n.a.	0.907	0.944 (0.362–2.464)	0.911	1.074 (0.309–3.738)	0.633	1.305 (0.438–3.890)	0.616	1.611 (0.250–10.395)
Having had COVID-19 (co: not having had COVID-19)	0.122	10.000 (0.539–185.465)	n.a.	n.a.	0.631	0.548 (0.047–6.350)	0.470	2.500 (0.208–30.039)	0.688	1.656 (0.141–19.477)	0.147	6.875 (0.507–93.141)
Someone in the social network having COVID-19 (co: no one in the social network having COVID-19)	0.191	3.167 (0.563–17.807)	0.508	2.750 (0.137–55.166)	0.609	1.310 (0.465–3.684)	0.840	1.147 (0.304–4.331)	0.225	0.429 (0.109–1.685)	0.580	0.528 (0.055–5.068)
Strong and very strong reduction in social network (co: no or weak reduction)	0.845	0.795 (0.081–7.858)	n.a.	n.a.	0.629	1.400 (0.357–5.487 =	0.841	0.816 (0.150–4.434)	0.772	1.277 (0.244–6.680)	0.626	0.560 (0.055–5.754)
Having malignant diseases (co: not having had a malignant disease)	0.268	3.500 (0.381–32.170)	0.648	0.500 (0.026–9.770)	0.150	2.143 (0.758–6.056)	0.503	1.622 (0.394–6.678)	0.524	0.695 (0.227–2.126)	0.646	1.700 (0,177–16.350)
PHQ2 ≥ 3	0.319	2.417 (0.426–13.713)	0.699	1.800 (0.091–35.424)	0.272	1.853 (0.617–5.565)	0.844	0.872 (0.224–3.391)	0.421	1.641 (0.503–5.350)	0.685	1.526 (0.198–11.780)
PHQ2 ≥ 5	0.896	1.167 (0.115–11.814)	0.265	6.000 (0.257–140.045)	0.145	3.522 (0.647–19.166)	0.535	0.500 (0.056–4.473)	0.513	1.667 (0.361–7.687)	n.a.	n.a.
GAD2 ≥ 3	0.613	0.541 (0.050–5.852)	0.265	0.167 (0.007–3.890)	1.000	1.000 (0.184–5.420)	0.478	0.523 (0.087–3.135)	0.245	0.381 (0.075–1.942)	n.a.	n.a.
GAD2 ≥ 5	0.869	0.825 (0.084–8.080)	n.a.	n.a.	0.124	0.365 (0.095–1.326)	0.197	0.243 (0.028–2.080)	0.636	0.708 (0.170–2.948)	n.a.	n.a.
PHQ4 ≥ 6	0.260	2.824 (0.463–17.210)	0.849	1.333 (0.069–25.912)	0.191	2.007 (0.706–5.707)	0.603	0.705 (0.189–2.628)	0.938	1.048 (0.326–3.368)	0.936	0.920 (0.120–7.076)
PHQ4 ≥ 9	0.980	0.971 (0.098–9.650)	0.144	13.000 (0.418–404.622)	0.451	1.806 (0.389–8.381)	0.452	0.433 (0.049–3.832)	0.713	0.731 (0.137–3.892)	n.a.	n.a.
BRS	0.070	0.284 (0.073–1.108)	0.299	0.007 (0.000–84.213)	**0.011**	**0.313 (0.128–0.764)**	0.524	0.742 (0.296–1.858)	0.282	0.634 (0.277–1.453)	0.328	0.492 (0.119–2.036)

BC = breast cancer; OC = ovarian cancer; GAD-2 = Generalized Anxiety Disorder Scale; PHQ-2 = Patient Health Questionnaire for Depression; PHQ-4 = Patient Health Questionnaire for Depression and Anxiety; BRS = Brief Resilience Scale; OR = odds ratio; CI = confidence interval; co = controls; n.a. = not applicable. Values in bold indicate statistical significance, as the level of statistical significance was set to p < 0.05.

To assess the independence of the most robust predictors of unfavorable lifestyle changes identified in the univariate analyses (p < 0.25), we performed a multivariate logistic regression analysis.

Age, living alone, having a history of BC or OC, GAD-2 ≥ 5, PHQ-2 ≥ 5, and resilience were found to be potential predictors of decreased PA during the COVID-19 pandemic. In the multivariate logistic regression model, only being at an increased risk for depressive disorder (PHQ-2 ≥ 5) emerged as an independent predictor (OR 12.719; 95 % CI 1.089–148.549; p = 0.043) for decreased PA during the COVID-19 pandemic. Based on 56 participants, this final regression model explained 18.1 % of the variance and exhibited 60.7 % sensitivity in predicting decreased PA.

In the multivariate logistic regression analysis, we did not identify independent predictors for other lifestyle changes, such as reduced efforts to maintain normal body weight, adherence to a balanced diet, avoidance of high-calorie foods intake, increased red meat consumption, or nicotine or alcohol consumption.

### 3.4 Changes in PA and prediction of incidence rates of BC

In order to estimate the potential excess in BC incidence resulting from decreased PA during the COVID-19 pandemic, we utilized a projection based on a previous prediction of 91,200 BC cases in women in Germany expected in the year 2030, which did not account for the pandemic's impact [36].

Assuming a latency period of 10 years between reduced PA and cancer incidence and using a RR = 0.90, which represents the difference in BC incidence rates between individuals with high versus low recreational PA [18], and a net percentage of 33.4 % of persons with no BC history and who practiced less PA during the pandemic, and assuming that their PA levels would not recover after the pandemic, we projected a total of 94,584 incident BC cases in Germany for the year 2030.

This estimation implies an absolute increase of 3384 BC cases due to changes in PA during the pandemic. To assess the potential economic impact, we considered the costs of medical treatment for BC at EUR 21,455 per patient in the first 11 months following diagnosis [37], and assumed that these expenses would remain stable for the next 10 years. The projected excess expenditure for the German healthcare system would amount to EUR 72,603,720.

## 4 Discussion

This study has uncovered noteworthy and concerning alterations in modifiable lifestyle factors within a vulnerable cohort of German women. A substantial proportion of the study population reported significant changes in their lifestyle patterns during the course of the COVID-19 pandemic. Notably, the most prominent changes were observed in the context of PA, with more than half of the participants indicating some form of alteration, be it a decrease or an increase in their level of PA. In contrast, changes in other lifestyle behaviors, such as the maintenance of a balanced diet or alcohol and nicotine consumption, were reported by fewer than half of the study participants.

These variations in lifestyle changes might be attributed to the extent of direct governmental intervention in specific routines, for instance, access to established sports facilities or participation in fixed sports groups. Such interventions could have been perceived as external barriers, extending beyond individual control. In contrast, other habits, such as daily dietary choices or smoking, were potentially regarded as more within the purview of personal responsibility. These findings highlight the complex interplay between external factors and individual agency in shaping lifestyle modifications during the pandemic.

A cross-sectional study in Germany, including the general population aged 14 years and above, was carried out between the end of March and the end of April 2020, revealing that 31.1 % of the study participants experienced a decline of PA, while 5.7 % reported an increase in PA [7]. In contrast, the present study has observed a substantial decrease in PA, with 41.6 % of participants reporting reduced PA during the first year of the pandemic. This divergence may be attributed to the unique characteristics of the study population under scrutiny. The current investigation focused on a vulnerable population at an increased risk of increased morbidity and mortality following infection with the SARS-CoV-2 virus [[3], [4], [5]]. Consequently, individuals in this cohort may have exhibited increased caution in their lifestyle choices, resulting in a more pronounced reduction of PA. This aligns with the findings of Engels et al. who noted that “fears of leaving the house” were more frequently reported by individuals who reduced their PA in Germany [40]. Additionally, our study assessed the decline in PA over the first year of the pandemic, not just during the initial month of lockdown, enabling us to analyze PA changes that occurred as the pandemic unfolded. Engels et al. demonstrated a decline in PA levels, including housework/gardening, sports and exercise, and active travel, during the initial and early stages of the second wave of the pandemic in German women [40].

The extended closure of sports facilities in Germany for over two months, beginning in late March 2020, may have disrupted established habits due to the measures aimed to mitigate the viral spread. Consequently, this may have led to the development of new behaviors that have the potential to compromise health. Similar patterns of change were reported in other populations as well [8,10,[41], [42], [43], [44], [45], [46], [47], [48]]. For instance, Salman and colleagues documented a decline in PA among 33.8 % of the female Kuwait population, whereas an increase in nicotine consumption was reported by only 4.3 % of female respondents [8]. A study of changes within Canadian families with young children observed a significant 59 % reduction in PA among female adult participants [42]. Among patients with cancer, 32 % reported a decrease in exercise, while 10 % engaged in increased PA during the pandemic [43]. Specifically, among BC patients, 38.5 % reported a decline in PA, while 12.8 % reported an increase [43]. Schoofs et al. in a large cohort study, reported that 53 % of the Dutch population studied experienced a decrease in PA during the early months of the pandemic [10].

In this study, we did not identify any significant and independent demographic or pandemic-specific predictors for the unfavorable changes in PA within our study population. A similar observation was made in a Dutch study, which found no noteworthy associations between PA and factors such as age, having children living at home, educational level, comorbidities, or resilience [10]. Conversely, some authors have reported age, shielding practices, employment status, and overall health status as predictors of lifestyle alterations in cancer patients during the pandemic [43,48]. Although an increased age, particularly beyond 51 years, showed a trend toward reduced engagement in PA in our study population, it did not reach statistical significance.

Prior research has documented behavioral changes in BC patients throughout their disease trajectory. Even before the pandemic, a decline in PA was observed in German women with BC during active cancer therapy, prompting suggestions for targeted interventions to restore recommended PA levels during survivorship [49]. The authors described a negative association between age and a decrease in PA, while a positive correlation was noted with active cancer treatment (chemotherapy or radiotherapy) and the presence of additional risk factors (such as hypertension or diabetes) in terms of PA decline [49]. Stress was identified as a barrier to healthy eating, and inaccessible fitness facilities were recognized as barriers to maintaining PA among women with BC before the pandemic [50,51]. Both stress and inaccessibility of fitness facilities coincided during the initial year of the pandemic and could have contributed to the observed lifestyle changes. Additionally, the pandemic exacerbated factors that had previously been acknowledged as barriers to engaging in PA [43].

A survey involving 1210 cancer patients in the United States, which investigates changes in PA during the COVID-19 pandemic (between August and September 2020), identified factors such as higher age at the time of the survey (mean 60 years; SD ± 11) and a self-reported health status rated as good to fair or poor (as compared to those reporting excellent health status) as variables associated with reduced PA [43]. No differences were observed between patient groups based on their COVID-19 infection status [43].

Women exhibiting an increased risk for depressive disorders, as identified by a PHQ-2 score ≥5, demonstrated a substantial and independent elevated risk, with a 12.7-fold greater likelihood of reduced engagement in PA after adjusting for potential confounding factors. It is noteworthy that even before the onset of the pandemic, individuals with depressive disorders were acknowledged to be at a higher risk of not adhering to recommendations regarding PA compared to those with other mental health conditions [52].

During the pandemic, our findings revealed a significant association between mental health, particularly depressive symptoms, and adverse alterations in modifiable lifestyle factors.

Studies in Spain, Sweden, and the UK confirmed that depressive symptoms were notably associated with decreased PA [48,53,54]. In contrast, anxiety did not show such a relationship [48]. Mental health played a dual role, acting as both a motivator and a hindrance to maintaining PA during the COVID-19 crisis. In Canada, reduced PA was credited to factors such as lack of motivation and restricted access to exercise facilities. However, the drive to maintain or increase PA was more about preserving mental health and well-being rather than physical appearance concerns [55].

This motivation shift may have also happened in the current study group. Thus, to maintain PA during future waves of the COVID-19 pandemic or other similar health crises, healthcare systems, and governmental authorities should consider providing guidance and education to vulnerable groups about the positive impact of PA on mental well-being, including its role in reducing anxiety, alleviating stress, and improving sleep patterns.

Healthcare professionals play a pivotal role in safeguarding the well-being of individuals by remaining vigilant of potential risk factors that may predispose individuals to adverse changes in modifiable risk factors. These professionals must regularly monitor, inform, and provide guidance to their patients on maintaining a healthy lifestyle. Additionally, healthcare professionals must stay well-informed about the latest guidelines and recommendations for lifestyle preventive measures. They should also be encouraged to proactively educate their patients, as previous research has revealed that not all healthcare providers are fully aware of current guidelines, and not every clinician considers it their responsibility to educate patients [56].

Notably, in the context of German cancer patients, structural obstacles have proven to be instrumental in preventing individuals from engaging in adequate PA. Surprisingly, the absence of informative materials was even more detrimental than the unavailability of sports facilities [57]. Thus, a structured and well-designed health-information campaign, which reaches the whole of society, and especially vulnerable groups, may be essential to prevent adverse health outcomes of the current pandemic or similar crises in the future. Consequently, a well-structured and comprehensive health information campaign, designed to reach all segments of society, with particular emphasis on vulnerable groups, may be deemed indispensable in averting adverse health consequences in anticipation of similar crises in the future.

Within the confines of this study population, a notable proportion, approximately 22.1 %, reported a heightened tendency toward unhealthy behavior concerning their adherence to a balanced diet and avoidance of high-calorie dietary choices. These observations are congruent with previously reported findings, where more than half of the participants in a Canadian study indicated changes in unhealthy dietary habits. At the same time, Italian students recorded an increase in the number of meals consumed [41,42]. Additionally, difficulties in maintaining healthy eating habits were documented in a cohort of Israeli individuals with chronic medical conditions, with over half of the study respondents reporting increased food intake compared to pre-pandemic levels [45].

Furthermore, 15.8 % of the individuals in this study population exhibited reduced efforts in achieving or maintaining a normal body weight. This trend could have profound implications, particularly within the group of women at an increased risk for BC or OC. It raises concerns as the shift in behavior may predispose individuals to the development of metabolic syndrome, increased susceptibility to cardiovascular diseases, and an elevated risk for various forms of cancer [9].

Our findings imply a significant interplay between unfavorable changes in health behaviors during the COVID-19 pandemic and their association with other potentially health-endangering practices. Conversely, healthy behaviors were linked with other health-promoting practices. These associations became evident through the strong and statistically significant correlations, such as the relationship between reduced efforts to maintain a balanced diet and increased consumption of nicotine or the decreased aim to achieve or maintain a normal body weight and a reduction of PA. Conversely, the correlations between reduced PA and adhering to a less healthy and balanced diet or striving for normal body weight, while statistically significant, exhibited weaker associations. This might be attributable to the multifaceted nature of PA regulation, influenced not only by individual intrinsic factors and health-related beliefs but also by extrinsic factors, particularly governmental-imposed lockdown measures, including the closure of sports facilities and the cancellation of group training sessions. Even before the pandemic, the lack of access to sports facilities has been acknowledged as a significant barrier to engaging in PA [22]. In accordance with our findings, Himbert and colleagues similarly reported an association between reduced exercise ans increased alcohol consumption during the pandemic. Conversely, cancer patients who adhered to other healthy lifestyle behaviors were more inclined to engage in increased PA [43].

Physical exercise plays a crucial role in preventing non-communicable diseases and certain malignancies. In this context, we propose the hypothesis of a potentially substantial increase in the incidence of BC in the aftermath of the COVID-19 pandemic. Previous studies have already reported an escalation in the burden of BC attributed to demographic shifts, including an aging population [36] and lifestyle factors [18,58]. To the best of our knowledge, this is the first study to estimate the increased burden of BC arising from lifestyle changes attributed to government-imposed social distancing measures and lockdowns, which were implemented to mitigate the viral spread.

The mechanisms that support the relationship between 10.13039/100006131PA and cancer development are various. PA exerts its influence on body mass, which in turn affects critical factors such as insulin resistance, the regulation of growth factors and steroid hormones, and the functioning of the immune system [21,23]. Notably, the body needs a relatively extended period to benefit from favorable adaptations induced by PA. Still, it takes only a few days of inactivity to reverse some of these positive changes. Even a brief period of reduced PA can increase inflammatory serum markers, and just three days of inactivity can induce insulin resistance. Furthermore, metabolic recovery may be a prolonged process, persisting even after the resumption of PA, particularly among elderly individuals [9,23].

The adverse changes in lifestyle factors that have manifested during the COVID-19 pandemic, potentially leading to an increased incidence of cancer and other non-communicable diseases, notably BC and colorectal cancer, may have far-reaching implications for global economies. The effects of government-imposed social distancing measures and lockdowns during the initial waves of the pandemic could result in substantially increased health expenditures for years and even decades to come, particularly if the detrimental lifestyle changes adopted during the pandemic persist.

To mitigate these potentially significant economic and health challenges, governments and healthcare systems worldwide should make substantial investments in information programs. These programs should emphasize the crucial health benefits associated with maintaining or reintroducing a healthy lifestyle, with a particular focus on promoting PA. This initiative aims to reduce the incidence and recurrence of various cancers, lower the risk of cardiovascular diseases and diminish the likelihood of adverse mental health outcomes [6,40].

Furthermore, it is imperative to develop and disseminate e-health-based exercise interventions that can be carried out at home. Recent research demonstrated their effectiveness in enhancing physical fitness, as exemplified in a study involving Spanish women with BC during the COVID-19 lockdown [59]. The population should also be reminded that 10.13039/100006131PA can potentially fortify the immune system to combat possible infections with SARS-CoV-2 or other infectious diseases while supporting the body's appropriate response to vaccinations against such viruses [6]. In addition to these efforts, society should make further investments in developing telemedicine lifestyle programs and home-based exercise regimens, specifically targeting vulnerable groups. This approach is essential in preparing for potential future pandemics [6].

## 5 Limitations

The findings of this study are primarily derived from an anonymous web-based survey conducted within patient support groups on 10.13039/100005801Facebook, which involved a relatively modest number of participants. The study targeted a specific group: women at increased risk of BC and OC, including those with a prior malignant diagnosis and those at increased risk without a history of malignant disease. This is a relatively specialized population, which may limit the number of available participants. Recruitment was conducted via 10.13039/100005801Facebook platforms of patient support groups. While social media can be an effective recruitment tool, the reach and effectiveness depend on the size of the groups, the level of engagement of group members, and the specificity of the criteria for inclusion. The method chosen is cost-effective and can access a wide geographic area, but it might not reach a large audience if the groups are small or if members are less active. Moreover, this approach likely increased the willingness of participants to respond but also limited the ability to follow up with potential participants who might have been interested but did not complete the survey. Next, participation was voluntary with no incentives provided, which can limit the number of respondents as incentives often increase participation rates. Consequently, the generalizability of the results may be limited. However, it is noteworthy that by the end of April 2021, approximately 4.03 % of the German population had been infected with the SARS-CoV-2 virus [60,61], a rate comparable with the infection rate among the study participants. This equivalence suggests that, at least concerning pandemic-related events, the study's findings may be considered representative of the German population.

It is essential to acknowledge that the recruitment of study participants exclusively through internet-based support groups may raise the possibility that the study population was less educated about healthy lifestyle practices than the general population. This discrepancy could potentially lead to an overestimation of the decline in PA or other unhealthy behaviors. Nevertheless, a study involving healthcare users in the United States with a history of BC highlighted the internet as the second most important source for gathering information about their condition [62]. Therefore, we assume that the study population of this study may be at least as well-informed about benefits of adhering to a healthy lifestyle as the general population. Moreover, support groups, whether professionally led or peer-led, play a substantial role in providing health education and promoting positive health behaviors among their members. They often offer guidance on physical exercise and dietary practices [63,64]. Consequently, it is reasonable to assume that the respondents in this study were better informed about healthy behaviors than the broader population.

Utilizing a cross-sectional design to assess lifestyle changes presents a constraint, as it limits our ability to track the evolution of these changes over time. Nevertheless, cross-sectional studies are a standard and robust method for determining the prevalence of health-related behaviors [41].

Another notable limitation arises from the reliance on self-reports and recollections of pre-pandemic PA. This methodology can introduce recall bias and socially desirable responses, potentially leading to underreporting, such as in the case of alcohol consumption. Furthermore, the study assessed PA changes without quantifying the PA amount. Previous research has indicated that self-reported current levels of PA tend to be overreported compared to directly measured PA [65]. Given that changes in life and lifestyle during the pandemic were consciously experienced by many, it is plausible that these changes were more readily recalled than in other settings. Additionally, the study did not differentiate between recreational and occupational PA, opting for a global estimation. It is likely that both forms of activity substantially decreased during the pandemic.

Lastly, it is essential to note that this study's estimated additional BC cases and potential excess healthcare costs are based on a heuristic approach. Consequently, future population-based studies should be conducted to comprehensively understand the pandemic's impact on cancer incidence and its economic implications for society.

## 6 Conclusion

The COVID-19 pandemic detrimentally impacted physical activity and other modifiable risk factors in a German group of women at risk for BC and OC. Evaluating mental health, particularly depression, can assist in identifying women prone to unfavorable lifestyle changes. While our findings may not be universally applicable, they warrant careful consideration due to the implications of lifestyle modifications on individuals, healthcare systems, and society, especially in the context of reduced PA resulting from pandemic-related measures.

It is imperative to inform and educate the population about regaining control over PA, adopting a balanced diet, preventing overeating, and embracing other healthy lifestyle practices to enhance self-efficacy and long-term health outcomes. We used mathematical modeling to project the impact of reduced PA during the pandemic on future BC incidence in Germany, offering a hypothesis-generating insight. Furthermore, our study can serve as a resource for counseling patients and individuals at risk on preventive measures for daily life during future pandemic waves or potential pandemics. Additionally, policymakers and healthcare systems should be aware of the possible long-term consequences of lockdown measures and develop strategies to sustain and promote healthy habits in anticipation of future pandemics or analogous crises. Longitudinal research is essential to comprehend the long-term effects of lifestyle changes within the general population.

In summary, our study underscores the necessity for measures that bolster and perpetuate a healthy lifestyle during similar scenarios or future pandemics, particularly among vulnerable groups.

## Ethics statement

This research was conducted anonymously and in strict adherence to ethical standards and principles in line with the Declaration of Helsinki and best clinical practices. All participants provided informed consent before their involvement in the study. Stringent measures were taken to ensure the privacy and confidentiality of the participants in compliance with applicable German and European laws and regulations. This survey was approved on January 20, 2021 by the local ethical review board of Rhineland-Palatinate, Mainz, Germany, approval number 15612.

## Funding

This research received no external funding.

## Informed consent statement

Informed consent was obtained from all subjects involved in the study.

## Patents

The authors have no patents related to this work.

## Data availability statement

The data used to support the findings of this study are included in the article.

## CRediT authorship contribution statement

**Kathrin Stewen:** Writing – review & editing, Writing – original draft, Methodology, Formal analysis, Conceptualization. **Annika Droste:** Writing – review & editing, Writing – original draft, Methodology, Data curation, Conceptualization. **Christian Ruckes:** Writing – review & editing, Methodology, Investigation, Formal analysis. **Tania Elger:** Writing – review & editing, Visualization, Data curation. **Susanne Theis:** Writing – review & editing, Visualization, Data curation. **Anne-Sophie Heimes:** Writing – review & editing, Visualization, Data curation. **Mona Wanda Schmidt:** Writing – review & editing, Visualization, Data curation. **Lina Judit Schiestl:** Writing – review & editing, Visualization, Software, Data curation. **Philip Herbert Klecker:** Writing – review & editing, Visualization, Software, Data curation. **Katrin Almstedt:** Writing – review & editing, Investigation, Data curation. **Marcus Schmidt:** Writing – review & editing. **Walburgis Brenner:** Writing – review & editing, Investigation. **Annette Hasenburg:** Writing – review & editing, Supervision. **Roxana Schwab:** Writing – review & editing, Writing – original draft, Visualization, Validation, Supervision, Resources, Project administration, Methodology, Investigation, Formal analysis, Data curation, Conceptualization.

## Declaration of generative AI and AI-assisted technologies in the writing process

During the preparation of this work the authors used ChatGPT and Grammarly in order to improve language and readability. After using this tool/service, the authors reviewed and edited the content as needed and take full responsibility for the content of the publication.

## Declaration of competing interest

The authors declare the following financial interests/personal relationships which may be considered as potential competing interests:Roxana Schwab reports a relationship with 10.13039/100004337Roche Pharma Schweiz AG Reinach that includes: non-financial support, speaking and lecture fees, and travel reimbursement. Roxana Schwab reports a relationship with 10.13039/100004325AstraZeneca AB that includes: non-financial support, speaking and lecture fees, and travel reimbursement. Roxana Schwab reports a relationship with 10.13039/100009947Merck Sharp & Dohme Corp that includes: speaking and lecture fees. Roxana Schwab reports a relationship with 10.13039/100019944Sanofi-Aventis Deutschland GmbH that includes: non-financial support, speaking and lecture fees, and travel reimbursement. Kathrin Stewen reports a relationship with 10.13039/100019944Sanofi-Aventis Deutschland GmbH that includes: speaking and lecture fees and travel reimbursement. Marcus Schmidt reports a relationship with 10.13039/100004325AstraZeneca AB that includes: board membership, consulting or advisory, funding grants, non-financial support, speaking and lecture fees, and travel reimbursement. Marcus Schmidt reports a relationship with 10.13039/501100022274Daiichi Sankyo Inc that includes: consulting or advisory, non-financial support, speaking and lecture fees, and travel reimbursement. Marcus Schmidt reports a relationship with 10.13039/501100003769Eisai Inc that includes: board membership, consulting or advisory, funding grants, non-financial support, speaking and lecture fees, and travel reimbursement. Marcus Schmidt reports a relationship with 10.13039/100004312Eli Lilly and Company that includes: board membership, consulting or advisory, non-financial support, speaking and lecture fees, and travel reimbursement. Marcus Schmidt reports a relationship with 10.13039/100009947Merck Sharp & Dohme Corp that includes: board membership, consulting or advisory, non-financial support, speaking and lecture fees, and travel reimbursement. Marcus Schmidt reports a relationship with 10.13039/100008792Novartis Pharma AG that includes: board membership, consulting or advisory, funding grants, non-financial support, speaking and lecture fees, and travel reimbursement. Marcus Schmidt reports a relationship with Pantarhei Bioscience BV that includes: board membership, consulting or advisory, funding grants, non-financial support, speaking and lecture fees, and travel reimbursement. Marcus Schmidt reports a relationship with 10.13039/100004319Pfizer Inc that includes: board membership, consulting or advisory, non-financial support, speaking and lecture fees, and travel reimbursement. Marcus Schmidt reports a relationship with 10.13039/100004337Roche that includes: board membership, consulting or advisory, non-financial support, speaking and lecture fees, and travel reimbursement. Marcus Schmidt reports a relationship with 10.13039/100020124Seagen Inc that includes: board membership, consulting or advisory, funding grants, non-financial support, speaking and lecture fees, and travel reimbursement. Marcus Schmidt reports a relationship with 10.13039/100004328Genentech Inc that includes: funding grants, non-financial support, speaking and lecture fees, and travel reimbursement. Marcus Schmidt reports a relationship with Palleos Healthcare that includes: funding grants, non-financial support, speaking and lecture fees, and travel reimbursement. Marcus Schmidt reports a relationship with 10.13039/100013226Pierre Fabre SA that includes: funding grants, non-financial support, speaking and lecture fees, and travel reimbursement. Annette Hasenburg reports a relationship with 10.13039/100004325AstraZeneca AB that includes: board membership, consulting or advisory, funding grants, non-financial support, speaking and lecture fees, and travel reimbursement. Annette Hasenburg reports a relationship with 10.13039/100006436Celgene GmbH that includes: consulting or advisory, funding grants, non-financial support, speaking and lecture fees, and travel reimbursement. Annette Hasenburg reports a relationship with GSK that includes: board membership, consulting or advisory, funding grants, non-financial support, speaking and lecture fees, and travel reimbursement. Annette Hasenburg reports a relationship with 10.13039/100004319Pfizer Pharma GmbH that includes: consulting or advisory, funding grants, non-financial support, speaking and lecture fees, and travel reimbursement. Annette Hasenburg reports a relationship with Tesaro Bio Germany GmbH that includes: board membership, consulting or advisory, non-financial support, speaking and lecture fees, and travel reimbursement. Katrin Almstedt reports a relationship with 10.13039/100004337Roche Pharma Schweiz AG Reinach that includes: funding grants, non-financial support, speaking and lecture fees, and travel reimbursement. Katrin Almstedt reports a relationship with 10.13039/100004319Pfizer Pharma GmbH that includes: funding grants, non-financial support, speaking and lecture fees, and travel reimbursement. Katrin Almstedt reports a relationship with 10.13039/100004325AstraZeneca 10.13039/100024877AB that includes: funding grants, non-financial support, speaking and lecture fees, and travel reimbursement. Anne-Sophie Heimes reports a relationship with 10.13039/100004319Pfizer Pharma GmbH that includes: funding grants, non-financial support, speaking and lecture fees, and travel reimbursement. Anne-Sophie Heimes reports a relationship with 10.13039/100004337Roche Pharma AG that includes: funding grants, non-financial support, speaking and lecture fees, and travel reimbursement. Anne-Sophie Heimes reports a relationship with 10.13039/501100022274Daiichi Sankyo Inc that includes: funding grants, non-financial support, speaking and lecture fees, and travel reimbursement. Marcus Schmidt has patent #EP 2390370 B1 licensed to Licensee. Marcus Schmidt has patent #EP 2951317 B1 licensed to Licensee. If there are other authors, they declare that they have no known competing financial interests or personal relationships that could have appeared to influence the work reported in this paper.
