# Supplementary material for: Changes in modifiable risk factors in women at increased risk for breast and ovarian cancer during the COVID-19 pandemic
Source: Heliyon. 2024 Jul 30;10(15):e35417. doi: 10.1016/j.heliyon.2024.e35417 (PMC11336576; doi:10.1016/j.heliyon.2024.e35417)
Supplement: Multimedia component 1 [file mmc1.docx]

Demographic characteristics

How old are you? [___] years

Do you live in a stable partnership? (*Yes – No*)

How would you describe your home/private environment?

- *Living alone (Yes – No)*
- *Living with children under 18 years* (*Yes – No*)
- *Living with older people (over 65 years)* (*Yes – No*)
- *Living with my spouse/life partner* (*Yes – No*)
- *others*

What is your highest educational qualification?

Are/were you infected by the SARS-CoV-2 virus yourself? (*Yes – No*)

Is/was someone in your environment infected with the SARS-CoV-2 virus? (*Yes – No*)

How much, on average, did you reduce your social contact network in the last 12 months due to the COVID-19 pandemic?

*Not at all – a little – moderate – significant – very much*

Questions about your risk of developing breast and/or ovarian cancer

To which risk group do you belong:

- I was diagnosed with a mutation in the BRCA1 or BRCA2 gene
- I was diagnosed with a different mutation (except BRCA1 or BRCA2 gene)
- I have an increased risk due to my family history, but I wasn’t diagnosed with a gene mutation (yet)

Are/were you already suffering from breast and/or ovarian cancer (benign tumors excluded)

*(multiple selection possible)*

- no, I am not/was not previously diagnosed with invasive breast and/or ovarian cancer or the respective premalignant lesions (in situ)
- yes, I am/was diagnosed with in situ breast lesions
- yes, I am/was diagnosed with in situ ovarian/tubal lesions
- I am/was diagnosed with invasive breast cancer
- I am/was diagnosed with invasive ovarian cancer

I. Substudy related to women’s expectations and opinions on health-related issues:

1. Are you concerned that if you become infected with the SARS-CoV-2 virus on your own, you might infect someone in your family or social network? (no concerns/ moderate concerns/ severe concerns/ does not apply)
2. Are you concerned that you may be susceptible to a more severe course of COVID-19 disease due to your increased risk of breast or ovarian cancer? (no concerns/ moderate concerns/ severe concerns/ does not apply)
3. Are you concerned, that infection with the SARS-CoV-2 virus may increase the risk of developing breast or ovarian cancer? (no concerns/ moderate concerns/ severe concerns/ does not apply)
4. If you have or have had breast and/or ovarian cancer, are you concerned about worsening of the oncological outcome resulting from an infection with the SARS-CoV-2 virus? (no concerns/ moderate concerns/ severe concerns/ does not apply).

II. Substudy related to lifestyle changes

How has your lifestyle changed in the last 12 months (during the global COVID-19-pandemic) in terms of:

1. Consumption of alcohol (significantly less - less -unchanged - more/increased – significantly more/increased - does not apply)

2. Smoking (significantly less - less -unchanged - more/increased – significantly more/increased - does not apply)

3. Exercising regularly (significantly less - less -unchanged - more/increased – significantly more/increased - does not apply)

4. Effort to achieve/maintain a healthy body weight (significantly less - less -unchanged - more/increased – significantly more/increased - does not apply)

5. Following a balanced diet and avoiding a high-caloric diet (significantly less - less -unchanged - more/increased – significantly more/increased - does not apply)

6. Avoidance of/reduction in red meat consumption (significantly less - less -unchanged - more/increased – significantly more/increased - does not apply)

III. Substudy related to opinion on hygiene measures in clinics during the covid-19-pandemic:

1. Would you have liked to be informed about hygiene protocols in advance of your appointment? (*Yes – No – I don’t know/does not apply)*

2. Would more information about the prevailing hygiene protocols have had a positive influence on your behavior (e.g., meeting appointments)? *Yes – No – I don’t know/does not apply*

3. Do you think that patients should be tested for SARS-CoV-2 infection before an ambulatory visit/appointment? *Yes – No – I don’t know/does not apply*

4. Do you think that medical personnel/physicians should be tested for SARS-CoV-2 infection on a regular basis? *Yes – No – I don’t know/does not apply*

5. Do you think that appointments should be scheduled in such a way to ensure that distancing rules can be strictly observed? *Yes – No – I don’t know/does not apply*

6. Should a relative or trustworthy person be allowed to accompany patients in the healthcare setting, despite the COVID-19 pandemic? *Yes – No – I don’t know/does not apply*

7. Do you think/agree that appointments which do not require one’s physical presence (e.g., counseling appointments) should be conducted as teleconferences or video conferences during the COVID-19 pandemic? *Yes – No – I don’t know/does not apply*

8. Do you think medical personnel should at least wear an FFP-1 mask (surgical mask) during the COVID-19 pandemic? *Yes – No – I don’t know/does not apply*

9. Do you think that medical personnel should always wear an FFP-2 mask during the COVID-19 pandemic to ensure patients’ safety? *Yes – no – I don’t know/does not apply*

IV. Substudy related to changes in care during the pandemic

How frequently are the following examinations/diagnostic measures usually conducted for you? Diagnostic measures (e.g., ultrasound of the breast gland, mammography, breast MRI) are aimed at diagnosing a tumor (or recurrence) in its early stages. Unfortunately, there are currently no examinations that can reasonably reliably detect the precursors of ovarian cancer or fallopian tube tumors. Nevertheless, in some women, repeated blood tests (e.g., tumor marker CA-125) or ultrasound examinations of the female internal genital organs (e.g., ovaries) are still conducted. Possible answers normally not performed, every 3 months, every 6 months, every 12 months, others, regarding:

- sonography of the breast
- mammography
- MRI
- Sonography of the lower genital organs
- CA-125
- others

Have the diagnostic examinations (e.g., for the early detection of breast cancer) taken place as planned in the last 12 months, or has the time interval between the respective examinations been changed by more than 1 month? Please answer the question regardless of whether you or the practice/clinic initiated the changes in scheduling. Possible answers: The examination is usually not performed/The examination was planned and could occur without significant delay (maximum +/- 1 month)/time frame between examinations shortened by at least one month/time frame between examinations prolonged by at least one month/examination was canceled regarding:

- sonography of the breast
- mammography
- MRI (normally not performed, every 3 months, every 6 months, every 12 months, others)
- Sonography of the lower genital organs
- CA-125
- others

In the last 12 months, was a risk-reducing surgery (i.e., removal of the healthy breast glands or healthy ovaries/fallopian tubes as a preventive measure) planned for you, and if so, was the scheduled timing adhered to?

Please answer this question regardless of whether you or the practice/clinic changed the scheduling. The operation was planned, and no, this operation was not planned.

The operation was planned and could take place without significant time delay (maximum +/- 1 month).

Possible answers: The operation was not planned/ The operation was planned and could take place without significant time delay (maximum +/- 1 month)./The operation was planned, and the scheduled date was postponed by more than 1 month/The operation was planned, but the scheduled date was moved forward by more than 1 month/The operation was completely cancelled, regarding:

- Risk-reducing removal of both breast glands (bilateral mastectomy)
- Risk-reducing removal of both breast glands (unilateral mastectomy)
- Risk-reducing bilateral salpingo-ovarectomy

Which therapy or surgery was planned for you in the last 12 months, and how has the treatment plan changed due to the COVID-19 pandemic? It is of interest whether there have been significant time shifts of MORE than 1 month (therapy/surgery either rescheduled 1 month earlier or postponed) as a result. Please answer the question regardless of whether you or the respective practice/clinic initiated the changes in treatment management.

Possible answers: The therapy or operation was planned and could take place without significant time delay (maximum +/- 1 month)/The therapy or operation was planned, and the start was delayed by more than 1 month/The therapy or operation was planned, and the start was moved forward by more than 1 month/The therapy or operation was stopped ahead of time/The therapy or operation was completely canceled regarding:

- Surgery
- Chemotherapy
- Immune therapy
- Radiation
- Endocrine therapy
- Others

What other measures were planned for you in the last 12 months, and how has the schedule changed due to the COVID-19 pandemic? It is of interest whether there have been significant time shifts of MORE than 1 month (therapy/surgery either rescheduled 1 month earlier or postponed) as a result. Please answer the question regardless of whether you or the respective practice/clinic initiated the changes in treatment management. Possible answers: planned and occurred without significant delay (+/- 1 month)/ start delayed by more than 1 month/start moved forward by more than 1 month/stopped ahead of time/completely canceled regarding:

- Genetic testing
- Rehabilitation
- Physiotherapy
- Lymphatic drainage
- Psycho(oncological) therapy
- Sexual therapy

If there have been the mentioned scheduling changes in the last 12 months (i.e., at least one of the planned appointments was rescheduled by more than 1 month either earlier or later), who initiated these changes? Multiple selections are possible if applicable.

- No scheduling changes occurred (by more than 1 month) for the planned diagnostic and/or therapeutic measures.
- I initiated the scheduling changes.
- The scheduling changes were initiated by the respective practice or clinic.

What reasons were decisive for you when you rescheduled the (previously mentioned) planned examination appointments/measures? Possible answers: I am not worried/I am moderately worried/ I am very worried/ does not apply regarding:

- Concern about contracting the coronavirus during the visit to the practice/clinic
- Concern about infecting other patients with the coronavirus during the visit to the practice/clinic
- Concern about infecting medical personnel with the coronavirus during the visit to the practice/clinic
- Concern about bringing the coronavirus home from a hospital/clinic visit and infecting family/housemates
- Concern about a higher risk of severe COVID-19 due to a known elevated risk of breast and/or ovarian cancer
- Concern about a higher risk of severe COVID-19 due to an existing or past tumor disease
- Had to cancel due to quarantine (contact with infected persons but not confirmed as ill)
- Had to cancel due to isolation (self-illness)
- I am not worried

What reasons were provided to you by the clinic/practice when they rescheduled the (previously mentioned) planned examination appointments/measures? In the free-text fields, you have the opportunity to specify other reasons not mentioned. Possible answers: No,Not True/ Yes,True/ Does Not Apply regarding:

- Reasons Provided by Clinic/Practice
- The reasons for the rescheduling were stated
- Due to the newly applicable hygiene rules during the COVID-19 pandemic, the number of patients had to be reduced
- Appointments were rescheduled due to clinic/practice closures
- Appointments were rescheduled/canceled due to a shortage of personnel
