# Supplementary figures and images for: Changes in modifiable risk factors in women at increased risk for breast and ovarian cancer during the COVID-19 pandemic
Source: Heliyon. 2024 Jul 30;10(15):e35417. doi: 10.1016/j.heliyon.2024.e35417 (PMC11336576; doi:10.1016/j.heliyon.2024.e35417)

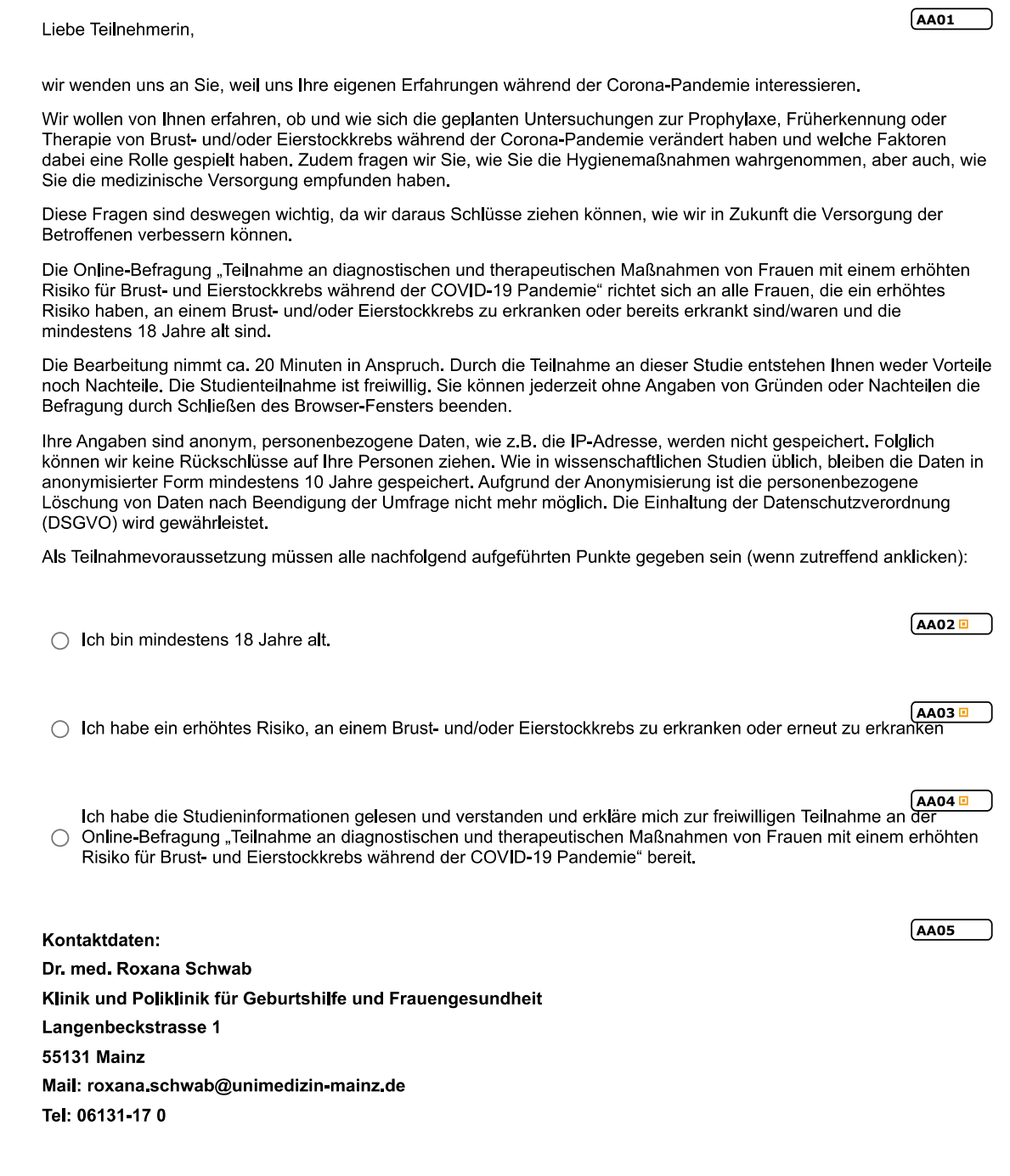

Supplement: Multimedia component 2 [file mmc2.docx]
